# Neuro-semantic prediction of user decisions to contribute content to online social networks

**DOI:** 10.1007/s00521-022-07307-0

**Published:** 2022-06-22

**Authors:** Pablo Cleveland, Sebastian A. Rios, Felipe Aguilera, Manuel Graña

**Affiliations:** 1 Business Intelligence Research Center, Universidad de Chile, Beauchef 851, P.O. Box 8370459, Santiago, Chile; 2 Industrial Engineering Department, Universidad de Chile, Beauchef 851, Santiago, Chile; 3 Computational Intelligence Group, University of the Basque Country, San Sebastian, Spain

**Keywords:** Multi-topic text preferences, Information diffusion, Leaky competing accumulator, Microscopic model of social interaction, Social interaction decision making

## Abstract

Understanding at microscopic level the generation of contents in an online social network (OSN) is highly desirable for an improved management of the OSN and the prevention of undesirable phenomena, such as online harassment. Content generation, i.e., the decision to post a contributed content in the OSN, can be modeled by neurophysiological approaches on the basis of unbiased semantic analysis of the contents already published in the OSN. This paper proposes a neuro-semantic model composed of (1) an extended leaky competing accumulator (ELCA) as the neural architecture implementing the user concurrent decision process to generate content in a conversation thread of a virtual community of practice, and (2) a semantic modeling based on the topic analysis carried out by a latent Dirichlet allocation (LDA) of both users and conversation threads. We use the similarity between the user and thread semantic representations to built up the model of the interest of the user in the thread contents as the stimulus to contribute content in the thread. The semantic interest of users in discussion threads are the external inputs for the ELCA, i.e., the external value assigned to each choice.. We demonstrate the approach on a dataset extracted from a real life web forum devoted to fans of tinkering with musical instruments and related devices. The neuro-semantic model achieves high performance predicting the content posting decisions (average *F* score 0.61) improving greatly over well known machine learning approaches, namely random forest and support vector machines (average *F* scores 0.19 and 0.21).

## Introduction

There is a huge amount of research literature dealing with diverse aspects of the analysis of on-line social networks (OSN). Classical research efforts are devoted to identify communities within the network [14, 39, 64], finding influencers or key members of the virtual community [6, 10, 29, 31, 34, 47, 63, 72], or describing the evolution of specific networks [25, 54, 62]. There is, however, very little or no work on the actual decision process conducting a user to publish some content in the OSN, e.g., posting a message in a forum of a virtual community of practice (VCoP). A VCoP implemented as an internet web-based Forum is a virtual place where members interact, discuss ideas, share, and generate knowledge about specific topics organized into sub-forums and discussion threads. Content generation is a radically different process from the propagation effects across the OSN that follow the publication of some new content. For instance, publishing a tweet is radically different from retweeting, sharing, liking, or any other propagation process that spreads the influence of the original tweet content. Synthetic content generation, such as *n*-gram Markov models allowing to generate fake tweets that are difficult to distinguish by humans [66], are out of the scope of the paper.

The decision to contribute a post to a discussion thread of a VCoP is a phenomenon affected by multiple factors like the user’s knowledge of the subject, his preferences, other users participating of the discussion, and even the quality of the information presented, among other factors. This decision process can be modeled by the competition of several simultaneously on-going threads to win the attention of the user, i.e., the user selects the winning thread for publishing a contribution. This competition is modeled by a neurophysiological model of choice, the leaky competition accumulator (LCA) [9, 76, 77], where the computational neurons activity is driven by a set of linear differential equations that accumulate inhibitive contributions from other neurons, excitatory input units, and fluctuations from and independent white noise source. LCA has been shown to account successfully for reaction time distribution empirically observed in psychophysical experiments. Specifically, for some combinations of parameter inhibition and decay values, LCA has been shown to reproduce the empirically observed violations of expected value and preference reversals reported in many experiments on value-based preferential choice. These studies focus on the distribution of the decision time for a fixed error ratio after many repetitions of the LCA run trying to mimic the distributions found empirically. LCA parameters are hand tuned (or explored in a grid search) in order to find the values that reproduce the desired response time behavior and the expected choice error ratio understood as choosing the lowest value option. Our work is more akin to machine learning approaches to model the decision process, i.e. we use LCA as decision making model whose performance is measured by the prediction accuracy of the decision made by the users to post a content contribution to a specific conversation thread where the semantic value assigned to the conversation thread is treated as a constant input.

For our specific work, we propose an extended LCA (ELCA) model in several aspects. First, the model includes many simultaneous choices by many users, while classical LCA considers a single agent and a small number of choices. Secondly, we use the semantic modeling of users and threads to compose the input value of each choice, thus linking the abstract valuation of the choices to concrete domain related evidences. Thirdly, we implement a genetic algorithm search for the ELCA model parameter calibration (aka training) using data from the content contribution decisions in a real life VCoP. The recovery of LCA parameters, stated as the induction of model parameters from simulation accumulator trajectories, has been acknowledged as an open difficult problem [49], which has been tackled by exploitation of Lie symmetries for a modified formulation of LCA equations [45]. Contrary to these approaches, we look for the optimal ELCA parameters that reproduce the actual user decisions after convergence of the simulation. However, our work does not try study or reproduce human choice phenomena, such as preference reversal, that are the original domain of study of the LCA model [9, 76, 77].

Semantic analysis of OSN published content is a current hot research area that allows to detect and prevent undesirable uses of the OSN. For instance, the semantic analysis at word level has been reported to allow to detect cyberbullying [30], helps detecting drunken tweets [24], and the age of users [56]. Also, social media posts content analysis allows to predict depression levels [2]. Specifically, we use unsupervised latent Dirichlet allocation (LDA) [8] topic analysis for the semantic modeling of the OSN published content, that allows to build up quantitative vectorial semantic representations of both users and conversation threads, not much unlike the social semantics neurobiological model based on conceptual knowledge [7]. LDA is a powerful tool that has been used to summarize and build network models of contents, such as semantic graphs relating publications about COVID-19 [1].

*Paper contributions and contents* This paper proposes a neuro-semantic model of the decisions made by the users to contribute contents to a VCoP web forum at the microscopic level. Specific contributions of this work are:

- The semantic characterization of the messages posted in the VCoP web forum is extracted by unsupervised formal topic analysis, namely LDA, allowing the semantic modeling of both users and conversation threads, so that user interest in generating content for a conversation thread can be quantified and assigned as an input value for the neurophysiological model of choice making, namely LCA.
- Ancillary information identifying key members of the social network provided by the online social network (OSN) administrators is used for the stratification of users improving the detail of the model of the content generation decision process.
- An extended LCA neurophysiological model of the user individual decision process to generate and contribute content in three ways: (1) use of semantically grounded value of the various choices, (2) the consideration of many choices and decision agents in a concurrent dynamic process, and (3) the estimation of the model parameters by maximizing prediction accuracy carried out by a genetic algorithm search. to the OSN that uses as input the semantic characterization of the users and the conversation threads.
- Prediction accuracy is based on a graph representation of the user contributions as a bipartite graph where nodes are either users or conversation threads, and edges correspond to the publication of a post by a user in a thread. Prediction performance measures are based on the distance between the ground truth graph extracted from the dataset and the predicted graph measured in terms of shared edges.

The paper is organized as follows: Sect. 2 presents related works on OSN information diffusion. Section 3 describes the materials and methods, including the description of the dataset, the semantic modeling, and the proposed neuro-semantic model for user content publication decisions. Section 4 reports the details and results of the computational experiments conducted. Finally, Sect. 5 gives our conclusions and future work directions.

## Related works

A great deal of the literature on OSN dynamic analysis has been focused the propagation of information across the network and the detection of communities and key influencer users. Table 1 gives a non-exhaustive summary of works found in the literature since 2007. There are two main research lines on models of information diffusion in networks [42], namely the explanatory and the predictive models. The first line of research includes modeling inspired in epidemics, while the second includes propagation models such as the cascade [20] or the linear threshold models [23]. This research is of utmost importance to areas like marketing, advertising, epidemiology, and social media analysis [79]. Some approaches to information spread modeling rely only on graph theory results [3, 71] assuming complete knowledge of the network, but they don’t report empirical validation over real data, some are purely speculative [27, 35, 52, 59, 69, 74, 81]. Aggregated predictions of macroscopic or mesoscopic behaviour of information diffusion have been also proposed [18, 26, 78–80]. For example, modeling the spread of information as epidemic propagation predicts the number of users that belong to the infected class [78–80] instead of trying to predict the individual infection. Other works model the density function of the distribution of influenced users [26], the node influence derived from the network topological properties [18], or the macroscopic information dissemination as the propagation of a signal over the network where interference between events is modeled by signal convolution [58]. At the microscopic level, learning from data the payoff of the social agents decisions allows accurate prediction of information diffusion [40]. Machine learning predictors of twitter activity have been developed [55], however data is not always available for confirmation of results. The role of topicality in Twitter adoption has been considered via machine learning predictive models [22] where topics correspond to selected hashtags, discovering that topicality plays a major role at microscopic information propagation. Hashtag topics are also used in the construction of the similarity measure underlying a radiation transfer model for influence prediction [5], but their role is not isolated.

**Table 1:** Information diffusion modeling approaches found in the literature

Ref./year	Model description	Results	Data set
[35]/2007	SIR model to estimate number of accesses to a site	N/A	“2 channel” web forum. DATA: number of posters per 15 min 9 p.m. Jan 10 2007–6 a.m. Jan 11 2007
[12]/2009	Topological properties of OSN graph	N/A	Flickr like data$^{1}$
[3]/2010	Game theoretic diffusion of technologies model that allows for competition between agents	N/A	Not applicable to implicit networks
[80]/2011	Topic-based SIR model. Applied to violent topic diffusion	*R*-square: 0.57–0.8	Ummah data set Dark Web Forum Portal by AI lab of U. of Arizona. 1,263,724 posts, 76,242 threads, 15,345 authors
[52]/2012	Probabilistic generative model of information emergence in networks, capturing internal and external exposures. URL diffusion	N/A	Tested on synthetic data and complete Twitter January 2011 data set. 3 billion tweets, 18,186 URLs
[81]/2012	SCIR model	N/A	Tested on synthetic data
[78]/2012	Event-driven SIR model	*R*-square: 0.66–0.89	Yahoo! Finance Walmart message board
[71]/2013	Deterministic model of competitive information diffusion on the Iterated Local Transitivity	N/A	Not applicable to implicit networks
[27]/2014	Evolutionary game theory model for diffusion dynamics	N/A	Twitter hashtag data set. 1000 Twitter hashtags, number of mentions per hour and time series
[74]/2014	SIS and SIR models with edge weights	N/A	Synthetic data
[69]/2015	Meme propagation model based on network topology	N/A	Tested on Higgs Twitter Network
[22]/2015	Adoption probability. Machine learning prediction	*F*1 = 0.93	Twitter hashtags and URLs 2009
[79]/2016	Topic-level SIR model	$R^{2}$ 0.52–0.75 and 0.44–0.79	Yahoo! Finance Walmart message board (139,062 threads, 441,954 messages, 25,500 authors) and US Politics Online Breaking News in Politics (2192 threads, 130,850 messages, 1124 authors)
[59]/2016	SIR model with stifling and forgetting mechanisms	N/A	Synthetic data and on OSN Renren (9590 nodes, 89,873 edges)
[26]/2017	Hydrodynamic information diffusion prediction model	$\overline{ACC}$: 76.2–88	6500 video tweets from Sina-weibo
[5]/2017	Physical radiation transfer	N/A	Twitter dataset about 9000 users
[40]/2017	Decision payoff modeling	Avg. precision: 0.7	Sina Weibo and Flickr datasets
[58]	Expectation maximizacion. Monte Carlo simulation	$R^{2}$: 0.98	SINA microblogging prediction of diffusion volume
[55]/2020	Bayesian logistic regression and random forests predictors	*F*1: 0.89–0.91	Twitter data crawled on informative and trending topics. N/A
[37]/2020	Modified forest fire	Num. spreaders	Twitter datasets

http://socialnetworks.mpi-sws.org/datasets.html

*N/A* not available

On the other hand, the semantic modeling of the information content published in the OSN is gaining attention. For instance, semantic analysis of social networks weibo and twitter based on single word topics has been applied to study the public perception on vaccines against COVID-19 [46]. It has been shown that semantic modeling of user contents allows for improved community detection [28, 82]. The impact of specific events on the social media can be assessed using semantic modeling. For instance, an approximate model [17] is shown to detect events in the social median, while event summarization on the basis of tweets can be achieved by a deep learning architecture [21]. Specifically, topic analysis by LDA has been used to uncover the meaning of events in social media [44] and the evolution of contents in the social media [15]. Notably, sentiment analysis has been proposed to predict song contest results [16]. For recommender systems, LDA-based topic hybrid recommender system has been proposed [33], and semantic analysis for recommendations has been also used in learning environments [32]. Moreover, semantic modeling of the user interactions with a chatbot allows for personalized interactions [43]. Semantic analysis may be extended in the time domain, allowing to measure changes in contents dynamically. Topic dynamics was applied to track the emergence of influential tweets about Fukushima disaster [53] over a long period of time. The consideration of both time and content allowed to monitor changes is a VCoP where the user exchange information about cosmetics [67].

## Materials and methods

### Computational pipeline

**Fig. 1:**
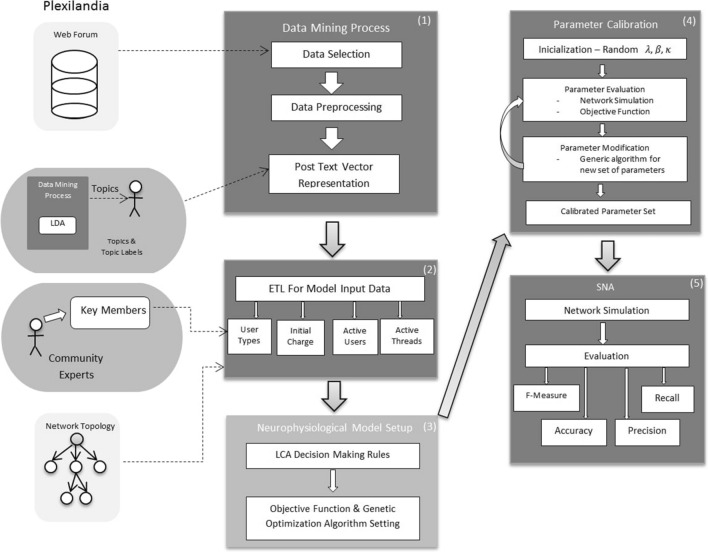
Study computational pipeline


The computational pipeline of this paper is shown in Fig. 1. It encompasses 5 phases corresponding to the numbered boxes in the figure going from left to right):

1. Data Mining Process: in this phase we carry out the curation and preprocessing of the raw OSN data described in Sect. 3.2. Section 3.3 describes data curation and preprocessing. Moreover we build a characterization of each forum contribution by LDA semantic unsupervised topic analysis. Section 3.4 gives a short overview of LDA.
2. Expert Training data Labeling (ETL): in this phase we prepare the user categorization using information from experts (i.e. the network administrators) as described in the Sect. 3.2. This categorization modulates some of the LCA parameters as discussed below.
3. Neurophysiological Model Setup: in this phase we formulate the LCA neural model that simulates the process of decision making for a content contribution published in some thread of a sub-forum. Our extended LCA (ELCA) is described in Sect. 3.6. From the LDA semantic model we construct the value of each conversation thread for each relevant user that will be the input for the ELCA contribution decision prediction. This construction is described in Sect. 3.5.
4. Parameter Calibration: We set up the genetic algorithm optimization to find the best parameter values of the neural model. The objective function is defined as the predictive performance over a subset of the dataset selected for model calibration. The genetic algorithm searches for the optimal settings of the LCA parameters using the data reserved for training. The genetic algorithm is described in Sect. 3.7.
5. Social Network Analysis (SNA) computational experiments: we apply LCA to simulate the content contribution decisions made by the users. The results of the simulation are used as prediction of the actual user behavior. The quality of the prediction is evaluated against the actual contributions registered for the time periods designed for validation. The predictive performance is measured by the *F*1 score. Experimental results are presented in Sect. 4.

An algorithmic description of the prediction of posts using the ELCA model is given in Algorithm 1, where the optimal values of the parameters $\hat{\beta _{c}}$ , $\hat{\kappa }_{c}$, and $\hat{\lambda }_{c}$ have been already estimated by the genetic algorithm that is described in Algorithm 2.

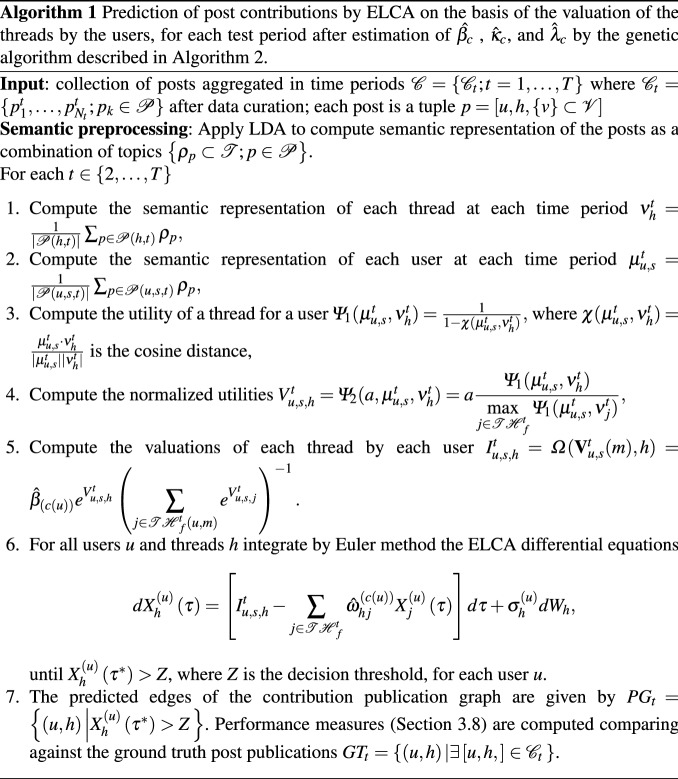

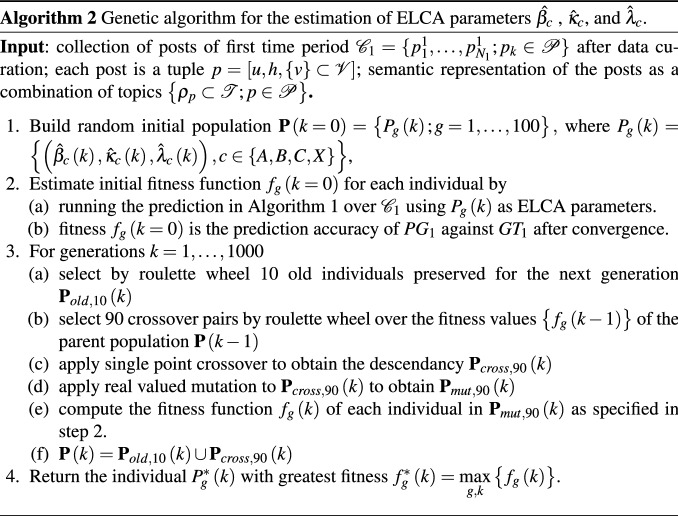


### Experimental dataset

The experimental works reported in this article are carried out over the data extracted from a web-based forum called *Plexilandia*, which was implemented as an OSN with more than 2500 members. *Plexilandia* supports a Virtual Community of Practice (VCoP) [6, 14, 62, 63, 65] specifically devoted to tinkering with musical apparatus that has been running for over 15 years. We have access to data from its greatest activity epoch, spanning 9 years. Table 2 contains the number of content publications *per* sub-forum along these 9 years, including the total number of posts. From now on, we may use the word “post” meaning a content contribution to a sub-forum.

**Table 2:** Plexilandia’s activity measured in number of content publications *per* relevant sub-forum *per* year

Sub-forum	2006	2007	2008	2009	2010	2011	2012	2013	2014	Total
Amplifiers (SF 2)	392	2165	2884	3940	3444	3361	2398	1252	985	20,821
Effects (SF 3)	184	1432	3362	3718	4268	5995	4738	2317	1331	27,345
Luthier (SF 4)	34	388	849	1373	1340	2140	926	699	633	8382
General (SF 5)	76	403	855	1200	2880	5472	3737	1655	1295	17,573
Pro Audio (SF 6)	–	–	–	–	–	342	624	396	219	1581
Synthesizers (SF 7)	–	–	–	–	–	–	–	104	92	196
**Total**	**686**	**4388**	**7950**	**10,231**	**11,932**	**17,310**	**12,423**	**6423**	**4555**	** 75,898**

Bold values correspond to summary values, either total or first order statistics, mean, min and max values

**Fig. 2:**
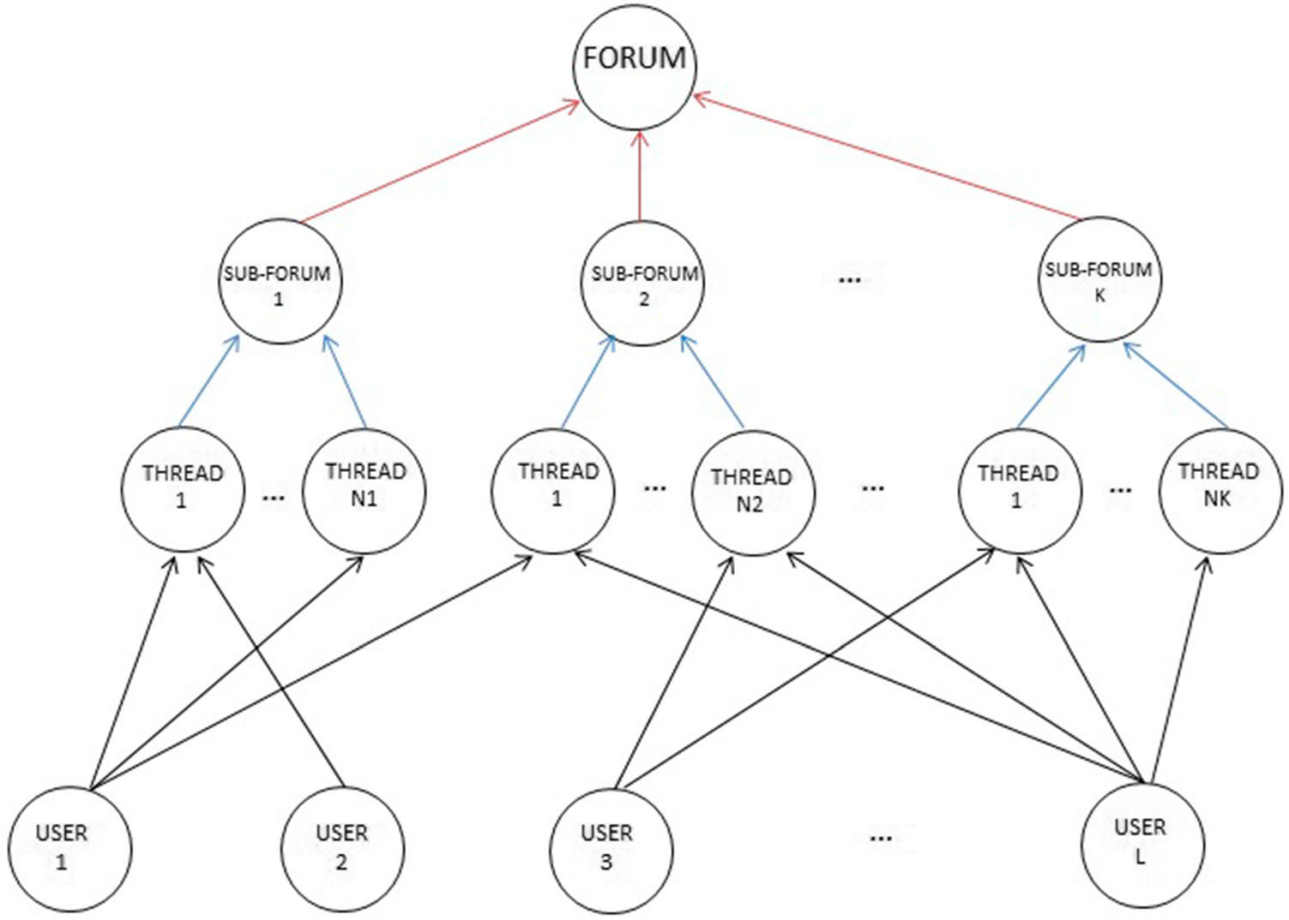
Hierarchical topology of VCoP web forums

The topics treated within Plexilandia’s forum are arranged into sub-forums according to the interest of the VCoP members that frequent it, namely Table 2 identifies the following sub-forums: Amplifiers, Effects, Luthiers, General, Audio for professionals, and Synthesizers. Contents published in such sub-forums should be strictly related to the purpose of the community, although spurious topics may emerge from unrestricted user interaction. The forum hierarchical structure of sub-forums is illustrated in Fig. 2.

Content contributions of users are conducted inside conversations that we will be denoting as *threads*. A thread about some discussion begins with a message posted by a user, containing a question or the presentation of an idea for discussion. Then, the different members of the community post their contributions thus increasing the shared knowledge about the central theme of the conversation. Each publication in the thread is composed of elements such as the user identifier (ID); the content contribution, which depending on the forum can be text, images, links to other pages, videos, and the management information of the forum system, such as publication creation date, the thread, and the topic it belongs to. All these elements might be taken into consideration but in this paper only the text content of posts will be exploited to build and analyze the social network.

#### Experimental training and validation data setup

**Fig. 3:**
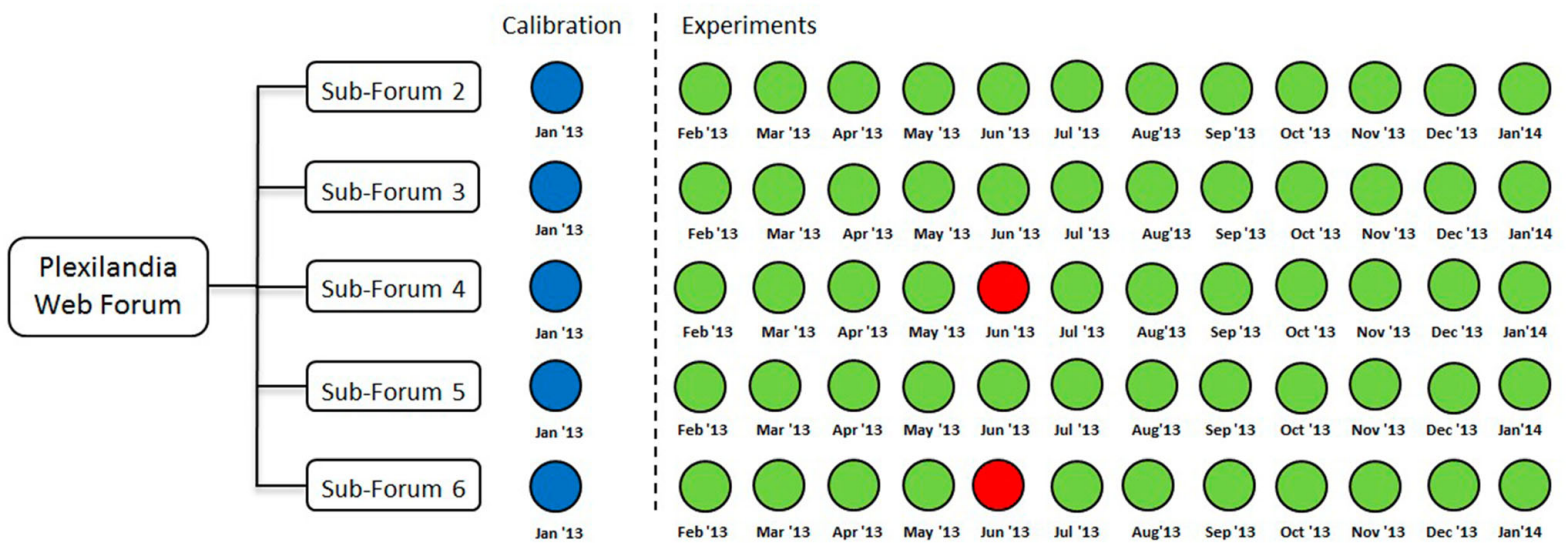
Experimental setup of data exploitation for model validation. Red dots correspond to months with missed data. Blue dots correspond to months whose data is used for training. Green dots correspond to months whose data is used for testing (color figure online)

According to the content structure of the Plexilandia Web Forum, the dataset is partitioned into sub-forums. For the computational experiments five sub-forums are considered. After examination of the distribution of the number of posts for different sizes of time periods (1 week, 2 weeks, 1 month, 2 months, 4 months) and the behavior of the threads during that time, a time period of 1 month has been selected, therefore aggregating the data into 13 time periods. The number of active users, active threads, and posts made during each of these 13 monthly time periods for each of the sub-forums is shown in Table 3. We provide an approximate ratio of imbalance (IBR) of each sub-forum computed as the number of possible content contributions, i.e. number of active users times the number of active threads, divided by the number of actual posts. Figure 3 shows the data partition for the validation experiments, using the data from the first month of 2013 (January) for the ELCA model calibration and the remaining months for testing. In other words, 8% of the data is used for the estimation of the optimal ELCA parameters by a genetic algorithm, and 92% for testing. Thus, model validation is set in the framework of training data scarcity, which is more realistic that training data abundance (such as when using 70% for training, 30% for testing) when trying to predict the online evolution of an OSN.

**Table 3:** Sub-forum statistics (number of active users, number of active threads, number of posts) *per* month

Month	Sub-forums				
	SF 2	SF 3	SF 4	SF 5	SF 6
1	(45, 25, 103)	(49, 43, 145)	(32, 40, 115)	(60, 37, 164)	(14, 11, 49)
2	(19, 10, 51)	(46, 29, 169)	(25, 8, 81)	(47, 27, 131)	(7, 5, 13)
3	(35, 20, 83)	(51, 46, 252)	(20, 13, 60)	(58, 30, 182)	(16, 6, 33)
4	(38, 27, 133)	(53, 43, 196)	(22, 15, 50)	(36, 23, 84)	(6, 5, 13)
5	(32, 22, 55)	(51, 44, 184)	(12, 8, 23)	(55, 28, 145)	(11, 9, 30)
6	(33, 22, 94)	(52, 38, 208)	(5, 3, 7)	(53, 36, 202)	(11, 5, 13)
7	(26, 14, 57)	(49, 32, 173)	(19, 10, 46)	(55, 35, 176)	(10, 7, 52)
8	(38, 24, 127)	(42, 37, 171)	(21, 17, 57)	(45, 29, 116)	(9, 3, 13)
9	(35, 17, 94)	(43, 33, 174)	(19, 10, 52)	(25, 19, 72)	(11, 7, 41)
10	(35, 23, 110)	(44, 29, 138)	(20, 9, 30)	(34, 25, 66)	(15, 5, 27)
11	(38, 22, 121)	(43, 24, 124)	(22, 9, 72)	(25, 13, 41)	(8, 5, 37)
12	(31, 19, 94)	(49, 38, 156)	(12, 8, 33)	(42, 25, 105)	(15, 6, 36)
13	(27, 14, 59)	(31, 30, 102)	(28, 17, 104)	(38, 24, 98)	(11, 6, 27)
**Total**	**(168, 221, 1181)**	**(174, 351, 2192)**	**(96, 134, 730)**	**(171, 282, 1582)**	**(501, 47, 384)**
IBR	31.43	27.86	17.6	30.48	61.32

Bold values correspond to summary values, either total or first order statistics, mean, min and max values

Last row contains the imbalance ration (IBR) computed as explained in the text

#### Categories of users

The OSN administrators provided a stratification of members for the year 2013 into four user categories [63] according to the role that they play in keeping the forum alive:

- Experts Type A: which are the most important key-members that create and sustain meaningful threads in relevant sub-forums. There are 34 such members based on administrators’ criteria.
- Experts Type B: which are also very important but to a lesser degree than A-type key-members. They contribute steadily but have less pivotal roles. There are 21 such members.
- Experts Type C: This type corresponds to those that are historic key-members. They have been involved in the social network since its origins, but they are not continuously participating. In this class, there are about 11 members.
- Non-experts or Type X: this class contains all members of the social network which are not key-members. They don’t belong to the social network core and usually, they ask questions rather than publish answers or tutorials.

We use only the data for the years 2013 and 2014 because we only have the information regarding key-members for these years [63]. We use the data of sub-forums 2 to 6. Discarding sub-forums 1 and 7 because they have not enough posts to contribute to the analysis.

### Data curation and preprocessing

The first step in our computational pipeline is the Plexilandia’s data curation and preprocessing [75]. First, we filter out the quotes from previous content contributions posted in the thread. A user can respond to a post by creating a new content contribution including a copy of the cited post plus the additional text of the new contribution. Therefore, it is necessary to delete the replicated part of the new post retaining only the new text input. Next, we transform the acronyms or abbreviations, eliminate spelling errors, and all elements of the posts that make them not comparable. This process is carried out by two natural language processing techniques: stemming and removing stop words. This serves to make posts comparable and to reduce the number of words used to compute post comparison. We apply LDA unsupervised topic modeling described in the next section for the semantic modeling of the content of documents [61].

### LDA topic analysis for semantic modeling

In this section we, give a brief account of the Latent Dirichlet Allocation (LDA) topic analysis used for semantic modeling. Let ${\mathscr {V}}$ be a vector of size $|{\mathscr {V}}|$ in which every row represents a different word used in the network, i.e. the vocabulary. Let $v_{i}$ be the word in place *i* of vector ${\mathscr {V}}$. It is possible to represent post $p_{j}$ as a sequence of $S_{j}$ words out of ${\mathscr {V}}$, with $S_{j}=|p_{j}|$, where $j\in \{1,\ldots ,|{\mathscr {P}}|\}$ and ${\mathscr {P}}$ corresponds to the number of posts that have been published in the VCoP forum. A *corpus* is defined as a collection of posts ${\mathscr {C}}=\{p_{1},\ldots ,p_{N}\}$. We can define the matrix ${\mathscr {W}}$ of size $|{\mathscr {V}}|\times |{\mathscr {P}}|$ where each element $w_{i,j}$ of this matrix is defined as the number of times the word $v_{i}$ appears in post $p_{j}$. Then $\sum _{i=1}^{|{\mathscr {V}}|}w_{i,j}=S_{j}$. Likewise, we can define $\sum _{j=1}^{|{\mathscr {P}}|}w_{i,j}=T_{i}$ which represents the total number of appearances of the term $w_{i}$ in the corpus.

A corpus can be represented by the product of the term frequency and the inverse document frequency (TF-IDF) matrix ${\mathscr {M}}$ of size $|{\mathscr {V}}|\times |{\mathscr {P}}|$ [68], which is defined as follows: each entry $m_{i,j}$ in the matrix is determined as

1 $$\begin{aligned} m_{i,j}=\frac{w_{i,j}}{T_{i}}\times \log \left[ \frac{|{\mathscr {P}}|}{1+n_{i}}\right] , \end{aligned}$$

where $n_{i}$ is the number of posts including the word $w_{i}$, $T_{i}$ is the maximum number of appearances of word $w_{i}$ in any post. The IDF term presented in Eq. (1) contains a correction with respect to the original IDF term $\log \left[ \frac{|{\mathscr {P}}|}{n_{i}}\right]$ to avoid undefined results when a post does not contain words after data curation. For dimension reduction we employ of an unsupervised topic discovery technique, namely, the LDA [4, 8] using the Gibbs sampling implementation [57]. This implementation does not search for the optimal values of the hyper-parameters $\alpha$, $\beta$, and number of required topics $|{\mathscr {T}}|=k$, so we have to make an empirical exploration to find them. LDA provides us with the distribution of each word over the discovered topics, the distribution of topics over the posts, and the *n* most important words that represent each topic together their belonging probabilities. In order to have fixed size probability vectors representing each topic $|{\mathscr {V}}|$, we pad them with zeros. These vectors are the columns of the semantic matrix (SM) $\left[ {\mathrm{Terms}}\times {\mathrm{Topics}}\right]$. In order to obtain the semantic description of the posts in a matrix of size $\left[ {\mathrm{Posts}}\times {\mathrm{Topic}}\right]$, we multiply the SM with ${\mathscr {M}}^{t}$, the transpose of the TF-IDF matrix defined by Eq. (1). The resulting $\left[ {\mathrm{Posts}}\times {\mathrm{Topic}}\right]$ matrix contains the semantic explanation of each post as a linear combination of the discovered topics via their vector semantic representations given by the rows of the matrix, denoted $\left\{ \rho _{p};p\in {\mathscr {P}}\right\}$.

### From semantic modeling to valuation

Let us denote ${\mathscr {U}}$, ${\mathscr {T}}\, {\mathscr {H}}$, and ${\mathscr {S}}\, {\mathscr {F}}$ the set of users, the set of threads, and the set sub-forums in the virtual community, respectively. The results of the LDA semantic analysis, namely the vectors $\rho _{p}$, allows to induce each user $(u\in {\mathscr {U}})$ multi-topic preference vector representation, and each thread ${\mathscr {T}}\, {\mathscr {H}}$ semantic content vector representation. The process to compute these semantic representations is as follows:

1. We aggregate the users content contributions according to the sub-forum ${\mathscr {S}}\, {\mathscr {F}}$ where they are posted.
2. We discretize the time axis into periods of size $\varDelta t$, thus creating a set of time periods *T*. Subsequently, we aggregate the content contributions from each sub-forum according to the time $(t\in T)$ period they belong to.
3. We extract the users (${\mathscr {U}}_{f}^{t}$) and threads (${\mathscr {T}}\, {\mathscr {H}}_{f}^{t}$) that are active during each time period. A user *u* is active in sub-forum *f* and period *t* if he makes a content contribution during this period. A thread *h* in sub-forum *f* is active if any user makes a content contribution to the thread during period *t*.
4. The thread semantic content vector representation for a period, denoted $\nu _{h}^{t}$, is the mean of the semantic vector representations $\rho _{p}$ for the content contributions that belong to both the thread *h* and the period *t*, formally:
  2 $$\begin{aligned} \nu _{h}^{t}=\frac{1}{|{\mathscr {P}}(h,t)|}\sum _{p\in {\mathscr {P}}(h,t)}\rho _{p}, \end{aligned}$$
  where $${\mathscr {P}}(h,t)=\{p\in {\mathscr {P}}:{{p}\, {\mathrm{is}}\,{\mathrm{posted}}\,{\mathrm{in}}\,{\mathrm{thread}}\, {h}\,{\mathrm{during}}\,{\mathrm{period}}\, {t}}\}$$.
5. To compute the user semantic representation, we categorize into subgroups, denoted *s*, the content contributions made by a user during a period according to the thread they were posted in. A user will have as many semantic vector representations for a period as threads that he has contributed to during this period. We denote the collection of these vector representations as $S_{u}^{t}$.
6. A user semantic vector representation for a period *t* and subgroup of content contributions *s*, denoted $\mu _{u,s}^{t}$, is the mean of the semantic vector representations $\rho _{p}$ for the content contributions made by the user *u* in this period of time, formally:
  3 $$\begin{aligned} \mu _{u,s}^{t}=\frac{1}{|{\mathscr {P}}(u,s,t)|}\sum _{p\in {\mathscr {P}}(u,s,t)}\rho _{p}, \end{aligned}$$
  where
  4 $$\begin{aligned} {\mathscr {P}}(u,s,t)&= \{p\in {\mathscr {P}}: {p}\, {\text {is}}\, {\text {posted}}\, {\text {by}}\,{\text {user}}\,{u}\nonumber \\ & \quad {\text {in}}\,{\text {period}}\, {t}\, {\text {and}}\,{\text { belongs}}\,{\text { to}}\, {\text {subgroup}}\, {s}\}. \end{aligned}$$

**Fig. 4:**
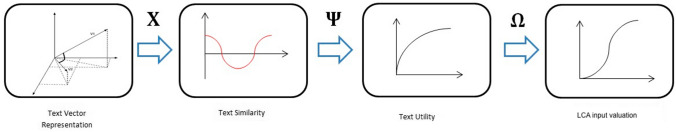
Transformations applied to the semantic modeling of users and threads to obtain the input values for the extended LCA

Now that we have the multi-topic semantic vector representation of the users and the semantic representation of the threads, we apply the computational pipeline shown in Fig. 4 to obtain the input for the extended LCA that implements the content contribution decision model.

1. First, we select a measure of the similarity $\chi$ of two semantic vector representations in the topic space. We use the cosine similarity, given by the cosine of the angle formed between two semantic vector representations. Thus, for a user multi-topic preference vector representation $\mu _{u,s}^{t}$ and a thread semantic content vector representation $\nu _{h}^{t}$, the similarity between them is given by
  5 $$\begin{aligned} \chi (\mu _{u,s}^{t},\nu _{h}^{t})=\cos (\theta )=\frac{\mu _{u,s}^{t}\cdot \nu _{h}^{t}}{|\mu _{u,s}^{t}||\nu _{h}^{t}|}, \end{aligned}$$
  where $\theta$ is the angle between $\mu _{u,s}^{t}$ and $\nu _{h}^{t}$.
2. Then, we define a function $\varPsi _{1}$ mapping semantic similarity into user utility. The utility that a user extracts from a thread is the expected number of times he chooses the thread over other threads to make a content contribution. Consider that $\pi =1-\chi (\mu _{u,s}^{t},\nu _{h}^{t})$ is the success probability parameter of a geometric distribution. Utility $\varPsi _{1}$ of the similarity between user and thread semantic representations is defined as follows [11]:
  6 $$\begin{aligned} \varPsi _{1}(\mu _{u,s}^{t},\nu _{h}^{t})=\frac{1}{1-\chi (\mu _{u,s}^{t},\nu _{h}^{t})}. \end{aligned}$$
  Furthermore, the preference of a user for a thread, i.e. the normalized user utility of a thread *h*, denoted $V_{u,s,h}^{t}$, takes into account all the threads in the sub-forum, computed by a function $\varPsi _{2}$ defined as follows:
  7 $$\begin{aligned} V_{u,s,h}^{t}=\varPsi _{2}(a,\mu _{u,s}^{t},\nu _{h}^{t})=a\frac{\varPsi _{1}(\mu _{u,s}^{t},\nu _{h}^{t})}{{ \max _{j\in {\mathscr {T}}\,{\mathscr{H}}_{f}^{t}}\varPsi _{1}(\mu _{u,s}^{t},\nu _{j}^{t})}}, \end{aligned}$$
  where parameter *a* modulates the preference of the users to threads whose topics are similar to the topics covered by the user content contributions. The greater the preference, the greater the satisfaction extracted from the conversation. Figure 5 plots an example of the utility values that a user attributes to the threads that are active at some period in time. Notice that only a few threads are of great interest to the user. Most active threads are stacked at the tail of the plot, meaning that they mostly contribute noise to the decision process. Therefore, we reduction in the number of alternative threads that a user takes into account during his decision-making process to generate content, keeping only the *m* threads with top utility values. This reduction of alternatives is based on classic research results about working memory and attention span [50].

**Fig. 5:**
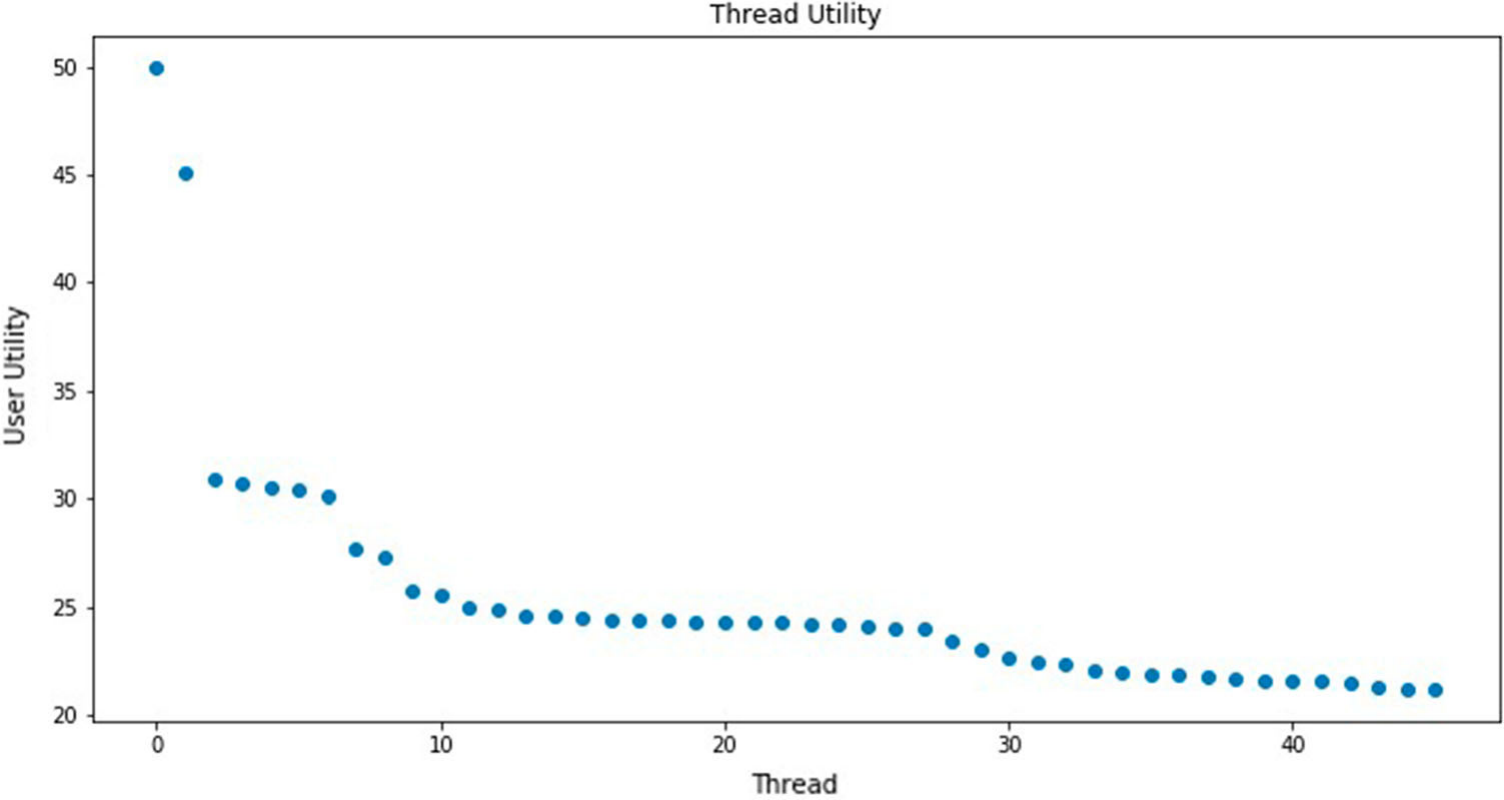
An instance of thread utility long tail distribution for a user at some specific time period
3. Finally, we define a function $\varOmega$ that maps the normalized user utility of each thread into the LCA input associated with the decision to make a content contribution to the thread, denoted $I_{u,s,h}^{t}$. For this purpose, we make use of random utility theory [11]: $I_{u,s,h}^{t}$ is proportional to the likelihood of choosing between alternative threads. Formally:
  8 $$\begin{aligned} I_{u,s,h}^{t}=\varOmega ({\mathbf {V}}_{u,s}^{t}(m),h)=\beta _{(c(u))}\frac{e^{V_{u,s,h}^{t}}}{{\sum _{j\in {\mathscr {T}}\,{\mathscr{H}}_{f}^{t}(u,m)}e^{V_{u,s,j}^{t}}}} \end{aligned}$$
  where $\beta _{(c(u))}$ is a proportionality parameter of the model that is specific for the category $c\left( u\right)$ of the user (defined as *A*, *B*, *C*, or *X* in Sect. 3.2), and ${\mathscr{T}}\,{\mathscr{H}}_{f}^{t}(u,m)=\{h\in {\mathscr{T}}\,{\mathscr{H}}_{f}^{t}:{{h}\,{\text{utility}}\, {\text{is}}\,{\text{one}}\, {\text{of}}\, {\text{the}}\,{\text{top}}\,{m}\,{\text{for}}\, {\text{user}}\, {u}}\}$.

### Extended leaky competing accumulator (ELCA)

**Fig. 6:**
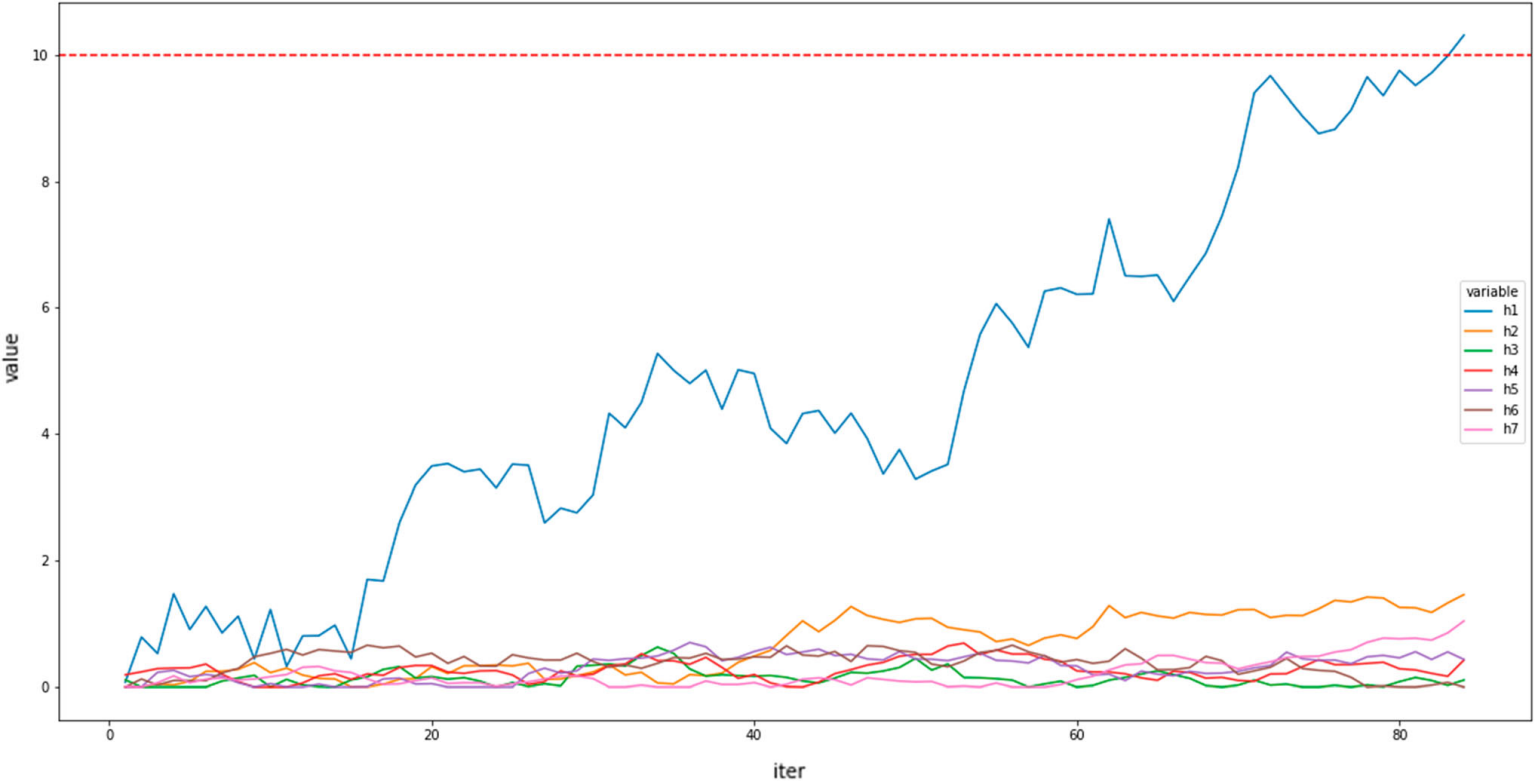
An instance evolution of the accumulators corresponding to a decision to post by a specific user

The decision process leading to the contribution of posts to conversation threads is modeled by an extended leaky competing accumulator (ELCA). The original LCA [9, 65, 76, 77] did only consider a decision carried out by a single agent, while our ECLA carries out simultaneously the decision processes of many users simultaneously, i.e., ECLA extends LCA over a community of users undertaking decisions simultaneously. We consider independent processes for each sub-forum *f* and each time period *t*. We define $X_{h}^{\left( u\right) }$ as the (neural) activation associated with the decision by user $u\in {\mathscr {U}}_{f}^{t}$ to publish a post in thread $h\in {\mathscr {T}}\,{\mathscr{H}}_{f}^{t}$. The decision process is implemented as dynamic process where the activation units evolve until one of them reaches a given threshold that triggers the corresponding decision. The evolution of the activation units for a user is illustrated in Fig. 6. Moreover, our ELCA has semantically grounded values associated to each choice, the term $I_{u,s,h}^{t}$ defined in Eq. (8), while classical LCA models have arbitrary values tuned by the researcher intuition. Finally, the provide a procedure to estimate the ELCA optimal parameters to reproduce the actual decisions made by the users, in a way similar to the training of conventional machine learning approaches.

The ELCA model describes the evolution of the joint decision process of all users as the simulation of the following set of dynamic stochastic equations:

9 $$\begin{aligned} {\mathrm{d}} X_{h}^{\left( u\right) }\left( \tau \right)&= \left[ I_{u,s,h}^{t}-\sum _{j\in {\mathscr {T}}\,{\mathscr{H}}_{f}^{t}}\omega _{hj}^{(c(u))}X_{j}^{\left( u\right) }\left( \tau \right) \right] {\mathrm{d}}\tau \nonumber \\&\quad +\sigma _{h}^{(u)}{\mathrm{d}}W_{h},\quad h\in {\mathscr {T}}\,{\mathscr{H}}_{f}^{t},u\in {\mathscr {U}}_{f}^{t}, \end{aligned}$$

that are integrated applying the Euler method. For each sub-forum *f* we have as many dynamic equations implementing concurrent decision processes as users and threads that are active during the time period *t*. The dynamic equations for each user *u* in Eq. (9) are integrated until time $\tau ^{*}$ when user *u* takes the decision to post a message to thread $h^{*}$, i.e. when the corresponding unit overcomes a decision threshold $X_{h^{*}}^{\left( u\right) }\left( \tau ^{*}\right) \ge Z$, as illustrated in Fig. 6. We have empirically set $Z=10$. Parameters $\omega _{hj}^{(c(u))}$ modulate the lateral inhibition by the other ongoing decision processes of user *u*, where $c\left( u\right) \in \left\{ A,B,C,X\right\}$ denotes the category of the user defined in Sect. 3.2. The term $I_{u,s,h}^{t}$ in Eq. (9) is an external constant input value in favor of posting a contribution in thread alternative *h* based on the semantic analysis developed above. Those input values are positive, i.e. $I_{u,s,h}^{t}\ge 0$. External input values are linearly accumulated in the activation variable $X_{h}^{\left( u\right) }$. It takes different values depending on the relation modeled and the category of the user, as shown in Eq. (10).

10 $$\begin{aligned} \omega _{ij}^{(c)}=\left\{ \begin{array}{cc} \kappa _{c} & i=j\\ \lambda _{c} & i\ne j \end{array}\right. ,\,\,\,c\in \left\{ A,B,C,X\right\} , \end{aligned}$$

where the $\kappa _{c}$ parameter models the activation decay of each unit [48]. Lateral inhibition between accumulator units is modeled by the $\lambda _{c}$ parameter. Equation (10) considers equal effect for all units stratified by the different user category defined by the OSN administrators. Following the biological inspiration, the activation variables are restricted to positive values ($X_{h}^{\left( u\right) }>0$). This hard limit has some interesting computational properties [9]. This model is in accordance with perceptual decision making [19]. Initial conditions $X_{h}^{\left( u\right) }(\tau =0)$ are specified by Eq. (11):

11 $$\begin{aligned} X_{h}^{\left( u\right) }(\tau =0)=(1+\gamma )^{l}-1 \end{aligned}$$

Parameter *l* in Eq. (11) denotes the number of times thread alternative *h* has been chosen previously, and parameter $\gamma \ge 0$ models the effect of repeated choices of the same alternative approaching the asymptotic curve defined in [38]. Recent works have shown convergence to a decision for large number of choices in a modified LCA model [45], but their model is limited to a single agent. They show that it is possible to recover the model parameters by maximum likelihood approach, however, they refer to the reproduction of simulation traces while we deal in the next section with parameter estimation to approximate the user decision behavior extracted from the real OSN data.

### ELCA parameter estimation by genetic algorithm

**Fig. 7:**
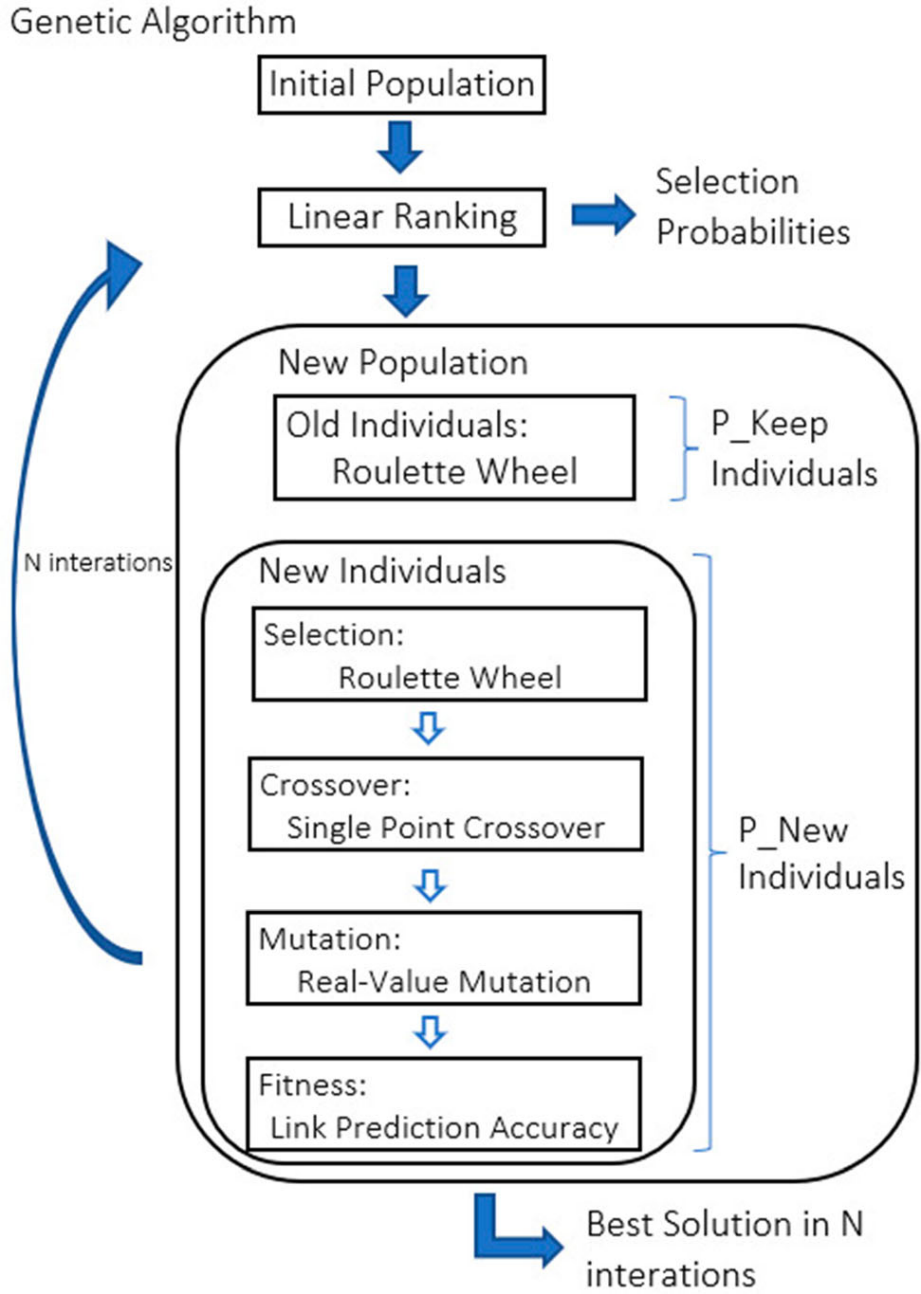
Flowchart of the GA used for ELCA optimal parameter search

ELCA parameter estimation was implemented by a genetic algorithm (GA) [73] illustrated in Fig. 7 with the following settings: Each individual $P_{g}\in {\mathbf {P}}$ in the GA population is composed of 12 real valued genes, which are estimations of the parameters of the LCA model for each kind of user in the sub-forum, i.e. $P_{g}=\left\{ \left( \hat{\beta }_{c},\hat{\kappa }_{c},\hat{\lambda }_{c}\right) ,c\in \left\{ A,B,C,X\right\} \right\}$. The size of the population was 100 individuals. The initial values of the individuals component parameters was generated following a uniform distribution in the [0, 1] interval. The fitness function is the accuracy of content contribution prediction by the LCA model using the individual parameter settings over the first month of the dataset. In other words, in order to compute the fitness of each individual in the population we run an instance of the LCA simulation comparing its track of post publication decision to the data from the first month. The individual selection for crossover is carried out by Baker’s linear-ranking algorithm [70] and roulette wheel selection [36]. Reproductive crossover was implemented by a single point crossover algorithm [60]. Mutation operator was a real-valued mutation [51]. Independent GA searches were carried out for each sub-forum. The details of the implementation, such as population size, number of generations computed, and the implementation of elitist selection policies are specified in Algorithm 2.

### Performance measures

As specified in Algorithm 1, the result of the ELCA simulation are user-thread pairs $PG_{t}=\left\{ \left( u,h\right) \left| X_{h}^{\left( u\right) }\left( \tau ^{*}\right) >Z\right. \right\}$ that are interpreted as predictors of the actual pairs that can be extracted from the ground truth post publications $GT_{t}=\left\{ \left( u,h\right) \left| \exists \left[ u,h,\right] \in {\mathscr {C}}_{t}\right. \right\}$. We make independent predictions for each time period and sub-forum. These pairs can be visualized as the edges of bipartite graphs that are the predicted and the ground truth publication graphs. We can define true positives as the edges that are in both graphs, true negatives as the edges that are absent from the two graphs, false positives are edges that appear in the prediction but are absent in the ground truth, and false negatives edges that are absent in the prediction but appear in the ground truth.

In order to evaluate the quality of the ELCA predictions, we compute 4 performance measures combining these basic measures. Namely: Recall, Accuracy, Precision, and the *F* measure. Recall is the ratio of true positives over the actual edges in the provided ground truth data:

12 $$\begin{aligned} {\mathrm{Recall}}=\frac{{{\mathrm{Number}}\,{\mathrm{of}}\,{\mathrm{true}}\,{\mathrm{positive}}\,{\mathrm{edges}}}}{{{\mathrm{Number}}\,{\mathrm{of}}\, {\mathrm{ground}}\,{\mathrm{truth}}\,{\mathrm{edgess}}}} \end{aligned}$$

Precision is the measure of specificity of the model, i.e. the probability of true positives predictions over all edge predictions made:

13 $$\begin{aligned} {\mathrm{Precision}}=\frac{{{\mathrm{Number}}\,{\mathrm{of}}\,{\mathrm{true}}\,{\mathrm{positive}}\,{\mathrm{edges}}}}{{{\mathrm{Number}}\,{\mathrm{of}}\,{\mathrm{predicted}}\,{\mathrm{edges}}}} \end{aligned}$$

*F* measure (aka $F_{1}$ score) combines precision and recall measuring the balance between them. It is defined as:

14 $$\begin{aligned} {F\,{\mathrm{measure}}}=\frac{2}{\frac{1}{{{\mathrm{Recall}}}}+\frac{1}{{{\mathrm{Precision}}}}} \end{aligned}$$

Accuracy is the measure of the overall true positive and negative link predictions:

15 $$\begin{aligned} {\mathrm{Accuracy}}=\frac{{{\mathrm{Number}}\,{\mathrm{of}}\,{\mathrm{true}}\,{\mathrm{positive}}\,{\mathrm{edges}}}+{{\mathrm{Number}}\,{\mathrm{of}}\,{\mathrm{true}}\,{\mathrm{negative}}\,{\mathrm{edges}}}}{{{\mathrm{Number}}\,{\mathrm{of}}\,{\mathrm{possible}}\,{\mathrm{edges}}}} \end{aligned}$$

Notice that, in our case study, the number of negative edges is much greater than the positive edges, hence the accuracy will be dominated by the prediction of negative edges, i.e. the absence of positive edge prediction, so that it can be high even if there are many missing actual edges. For this reason, we focus the report of results on the *F* measure that is a more trustful measure in case of high class imbalance.

## Results and discussion

### Experimental results

As described in Fig. 3, for each sub-forum we carry out an independent GA search to obtain the optimal parameters for the ELCA model over the data from month 1. The optimal ELCA parameter values obtained by the GA search for each sub-forum are specified in Table 4. The ELCA model with these parameter settings is used to predict the generation of posts from users on specific threads for each sub-forum and for each month between February 2013 and January 2014. The average prediction performance results of the ELCA approach are given in Table 5. In Table 6, we present the detailed results in terms of the *F*-measure for each sub-forum and for each month considered within the time frame. The overall mean *F*-measure score of ELCA across all sub-forum experiments is 0.61.

**Table 4:** Optimal ELCA parameter values for each sub-forum found by independent GA searches over the training data (January 2013)

	${\beta _{\mathbf{A}}}$	${\beta _{\mathbf{B}}}$	${\beta _{\mathbf{C}}}$	${\beta _{\mathbf{X}}}$	${\kappa _{\mathbf{A}}}$	${\kappa _{\mathbf{B}}}$	${\kappa _{\mathbf{C}}}$	${\kappa _{\mathbf{X}}}$	${\lambda _{\mathbf{A}}}$	${\lambda _{\mathbf{B}}}$	${\lambda _{\mathbf{C}}}$	${\lambda _{\mathbf{X}}}$
Sub-forums												
SF 2	0.863	0.148	0.511	0.553	0.174	0.055	0.070	0.965	0.491	0.137	0.399	0.189
SF 3	0.584	0.906	0.389	0.029	0.684	0.340	0.217	0.588	0.146	0.951	0.189	0.949
SF 4	0.586	0.833	0.352	0.476	0.642	0.389	0.866	0.981	0.639	0.478	0.107	0.245
SF 5	0.628	0.184	0.000	0.429	0.707	0.733	0.047	0.623	0.0935	0.864	0.847	0.640
SF 6	0.516	0.126	0.490	0.595	0.287	0.692	0.087	0.401	0.956	0.869	0.044	0.315

**Table 5:** Predictive performance results averaged over all test periods of the proposed ELCA approach *per* sub-forum

	Sub-forums				
	SF 2	SF 3	SF 4	SF 5	SF 6
Mean recall	0.55	0.48	0.63	0.51	0.83
Mean accuracy	0.92	0.93	0.89	0.93	0.92
Mean precision	0.57	0.50	0.67	0.53	0.85
Mean *F*-measure	0.56	0.49	0.65	0.52	0.84

**Table 6:** Detailed *F*-measure results of the proposed ELCA *per* testing month and sub-forum

Month	2	3	4	5	6	7	8	9	10	11	12	13	Mean	Max	Min
Sub-forums															
SF 2	0.72	0.54	0.45	0.52	0.49	0.65	0.54	0.58	0.58	0.49	0.53	0.68	**0.56**	**0.72**	**0.45**
SF 3	0.44	0.47	0.51	0.44	0.50	0.44	0.47	0.45	0.56	0.43	0.48	0.65	**0.49**	**0.65**	**0.43**
SF 4	0.65	0.48	0.63	0.78	***	0.61	0.67	0.66	0.71	0.68	0.72	0.55	**0.65**	**0.78**	**0.48**
SF 5	0.49	0.44	0.56	0.46	0.39	0.47	0.47	0.68	0.63	0.65	0.51	0.45	**0.52**	**0.68**	**0.39**
SF 6	0.82	0.81	0.86	0.84	***	0.84	0.80	0.92	0.95	0.85	0.84	0.69	**0.84**	**0.95**	**0.69**

Bold values correspond to summary values, either total or first order statistics, mean, min and max values

*Comparison with machine learning approaches* For comparison, we have carried out the training of conventional machine learning approaches. The dataset for training is extracted from the same period (first month) used to calibrate the ELCA model. For each possible pair of active user *u* and thread *h*, we define the feature vector concatenating the semantic descriptions of the user and the thread ${\mathbf {x}}_{u,h}=(\mu _{u,s}^{t},\nu _{h}^{t})$, and the class variable $y_{u,h}\in \left\{ {\mathrm{existing}},{\text{non-existing}}\right\}$ that signals if there is at least one post by user *u* in thread *h* in this time period. The testing data are composed of similar feature vectors from the remaining time periods. We have tested two well know algorithms using conventional implementations provided in Matlab. First, a random forest (RF) with 101 individual trees. Secondly, a linear support vector machine (SVM).

Tables 7 and 8 give the detailed *F*-measure results for the RF and SVM. The overall average of the *F*-measure of the RF and SVM predictors over all sub-forum experiments are 0.19 and 0.21, respectively, far below the average result achieved by our ELCA approach (0.61). The best *F* score result for a specific month and sub-forum of ELCA (0.95) is far above that of RF (0.60) and SVM (0.63). A one sided Wilkoxon’s rank sum test comparing the entries of Table 6 against Tables 7 and 8 confirms that the superiority of the ELCA model is extremely significative ($p<1e{-}16$).

**Table 7:** Detailed *F*-measure results of the Random Forest approach *per* testing month and sub-forum

Month	2	3	4	5	6	7	8	9	10	11	12	13	Mean	Max	Min
Sub-forums															
SF 2	0.20	0.16	0.10	0.14	0.13	0.17	0.10	0.15	0.14	0.12	0.13	0.17	**0.14**	**0.20**	**0.10**
SF 3	0.08	0.07	0.09	0.07	0.08	0.12	0.10	0.10	0.11	0.15	0.08	0.14	**0.10**	**0.15**	**0.07**
SF 4	0.22	0.19	0.24	0.40	***	0.30	0.19	0.22	0.27	0.26	0.31	0.17	**0.23**	**0.40**	**0.17**
SF 5	0.13	0.10	0.11	0.11	0.07	0.08	0.09	0.14	0.14	0.22	0.10	0.11	**0.11**	**0.22**	**0.07**
SF 6	0.43	0.30	0.55	0.30	***	0.29	0.59	0.32	0.36	0.35	0.60	0.28	**0.38**	**0.60**	**0.29**

Bold values correspond to summary values, either total or first order statistics, mean, min and max values

**Table 8:** Detailed *F*-measure results of the SVM approach *per* testing month and sub-forum

Month	2	3	4	5	6	7	8	9	10	11	12	13	Mean	Max	Min
Sub-forums															
SF 2	0.21	0.19	0.11	0.12	0.11	0.13	0.11	0.17	0.15	0.18	0.11	0.15	**0.145**	**0.21**	**0.11**
SF 3	0.10	0.05	0.11	0.09	0.10	0.15	0.12	0.13	0.16	0.19	0.11	0.13	**0.12**	**0.19**	**0.05**
SF 4	0.18	0.22	0.28	0.38	***	0.33	0.22	0.19	0.23	0.25	0.28	0.18	**0.25**	**0.38**	**0.18**
SF 5	0.11	0.13	0.15	0.11	0.11	0.10	0.11	0.13	0.17	0.26	0.12	0.16	**0.14**	**0.22**	**0.11**
SF 6	0.39	0.31	0.45	0.31	***	0.25	0.61	0.33	0.39	0.33	0.63	0.27	**0.39**	**0.63**	**0.25**

Bold values correspond to summary values, either total or first order statistics, mean, min and max values

****entries correspond to non convergent computation processes, i.e. we do not reach a final value

### Discussion

**Fig. 8:**
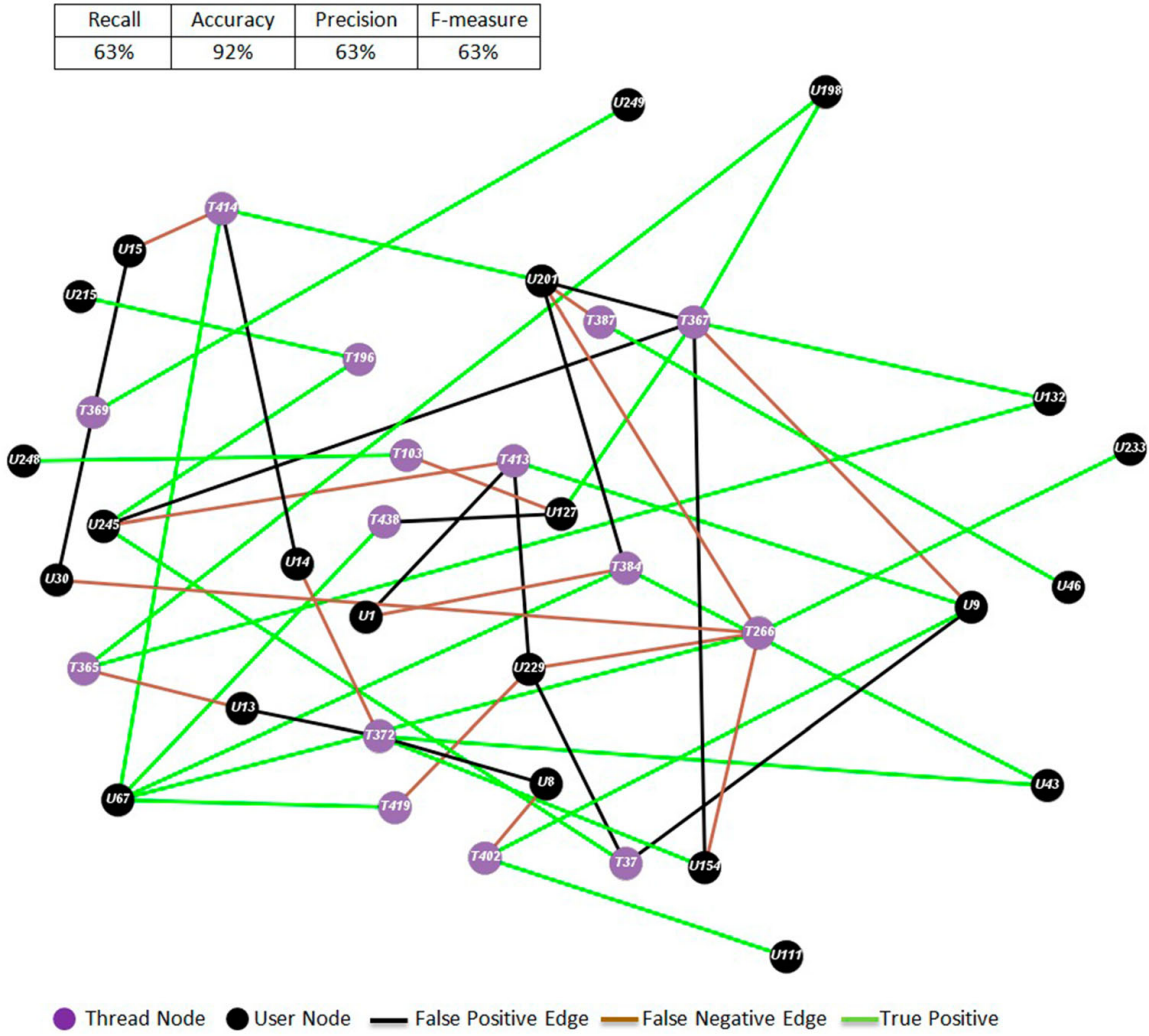
Example of middle performance result corresponding to the post publication graph of SF 4 for Month

**Fig. 9:**
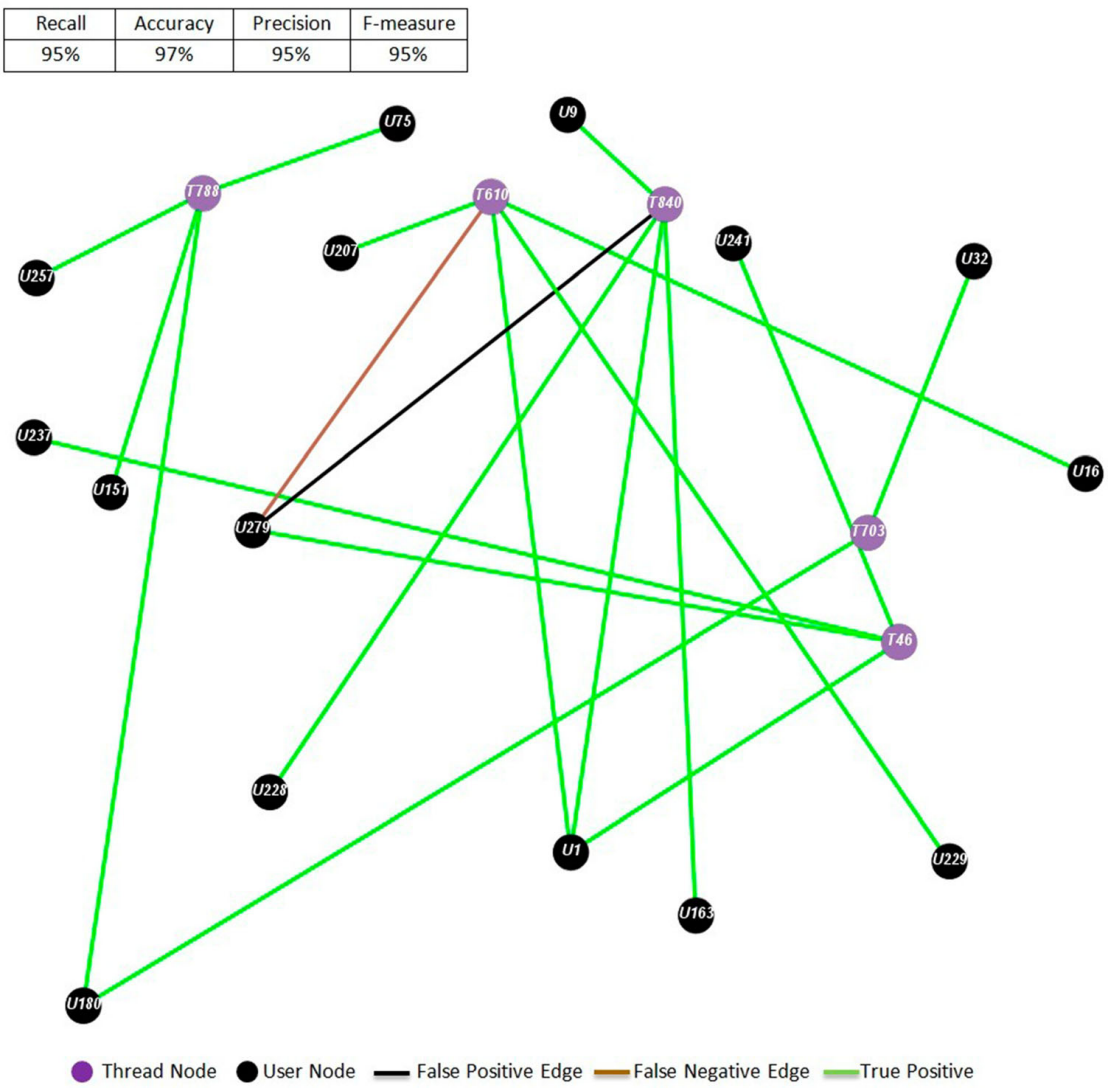
Best predictive performance corresponding to post publication graph of SF 6 for Month 10

For a qualitative appreciation of the results, Figs. 8 and 9 show the graph representations of the content publication predictions for sub-forum 4 at month 4 and sub-forum 6 at month 10, where violet and black nodes correspond to threads and users, respectively. Green edges correspond to the content contributions that the ELCA simulation predicted correctly, black edges are false positives, and brown edges correspond to false negatives. Tables 9 and 10 display the content publishing rules derived from the ELCA simulation. We can notice that most of the network edges are green and that there is approximately the same amount of predicted edges and ground truth edges, which is a very important structural property we must comply with. There are few false positives compared to the large number of non-existing links. This is the reason for the high values of the accuracy performance measure in Table 5 relative to the other measures which only take into account the true positives. We recall from Table 3 that our sub-forum datasets can be considered as very imbalanced two class datasets if we aim to predict the links between users and threads. It is well known, that most classifiers are biased towards the majority class (here the non-existing links). Undersampling the majority class or over-sampling the minority class are proposed as means to improve the performance on the minority class, however it is not clear how to carry out these procedures over our sub-forum data.

**Table 9:** Post publication decision rules for SF4-M4

User	Posts in:	User	Posts in:	User	Posts in:
U1	T384, T413	U46	T387	U215	T196
U8	T372, T402	U67	T266, T384, T414, T419, T438	U229	T37, T266, T413, T419
U9	T37, T367, T402, T413	U111	T402	U233	T266
U13	T365, T372	U127	T103, T367, T438	U245	T37, T196, T367, T413
U14	T372, T414	U132	T365, T367	U248	T103
U15	T369, T414	U154	T266, T367, T372	U249	T369
U30	T266, T369	U198	T365		
U43	T372, T384	U201	T266, T367, T384, T387, T414		

User = U**, conversation threads the user has published posts in = T***

**Table 10:** Post publication decision rules for SF6-M10

User	Posts in:	User	Posts in:	User	Posts in:
U1	T46, T610, T840	U151	T788	U229	T610
U9	T840	U163	T840	U237	T46
U16	T610	U180	T703, T788	U241	T46
U32	T703	U207	T610	U257	T788
U75	T788	U228	T840	U279	T46, T610, T840

User = U**, conversation threads the user has published posts in = T***

**Fig. 10:**
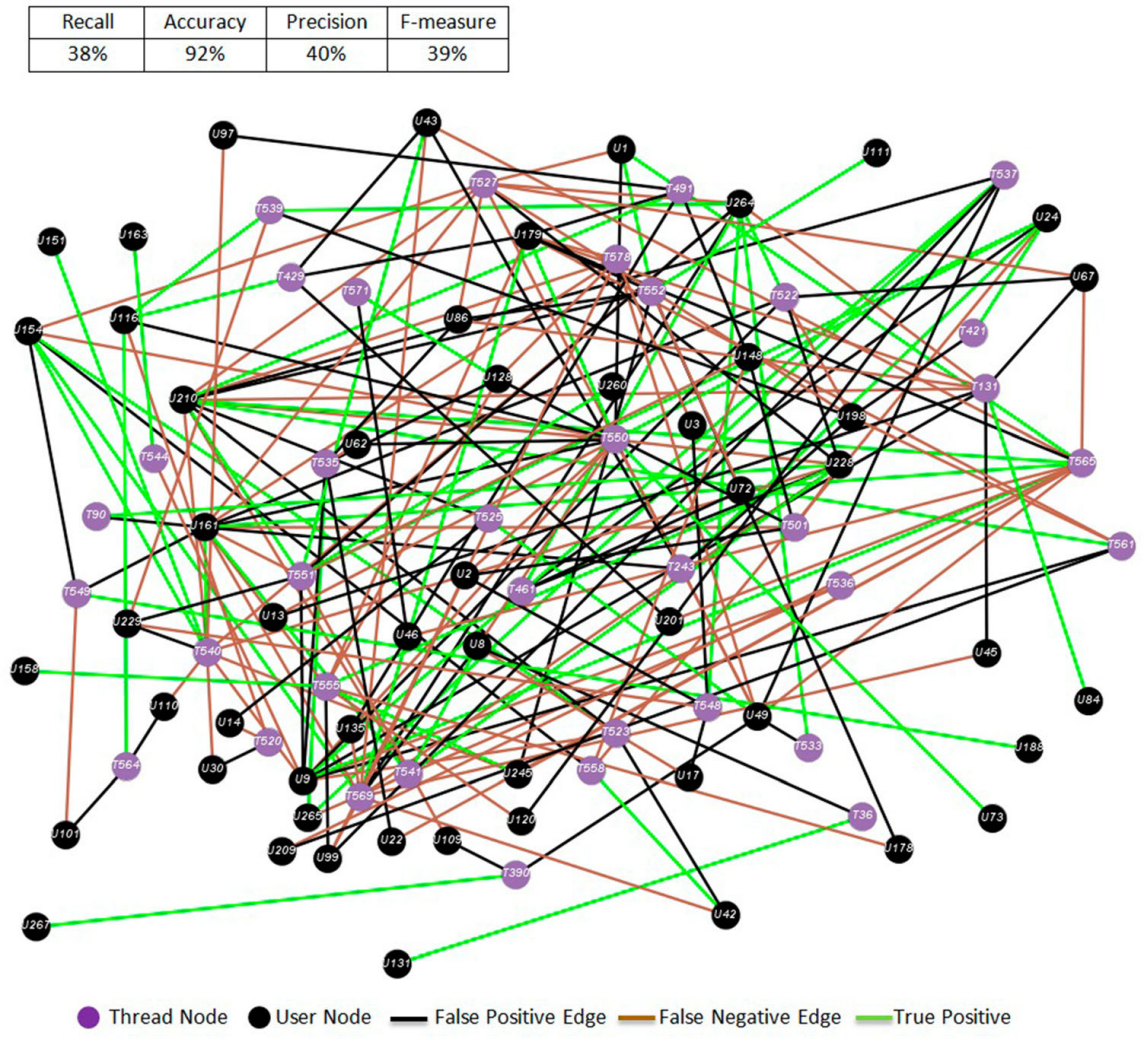
Worst result corresponding to publication graph of sub-forum 5 for Month 6

**Fig. 11:**
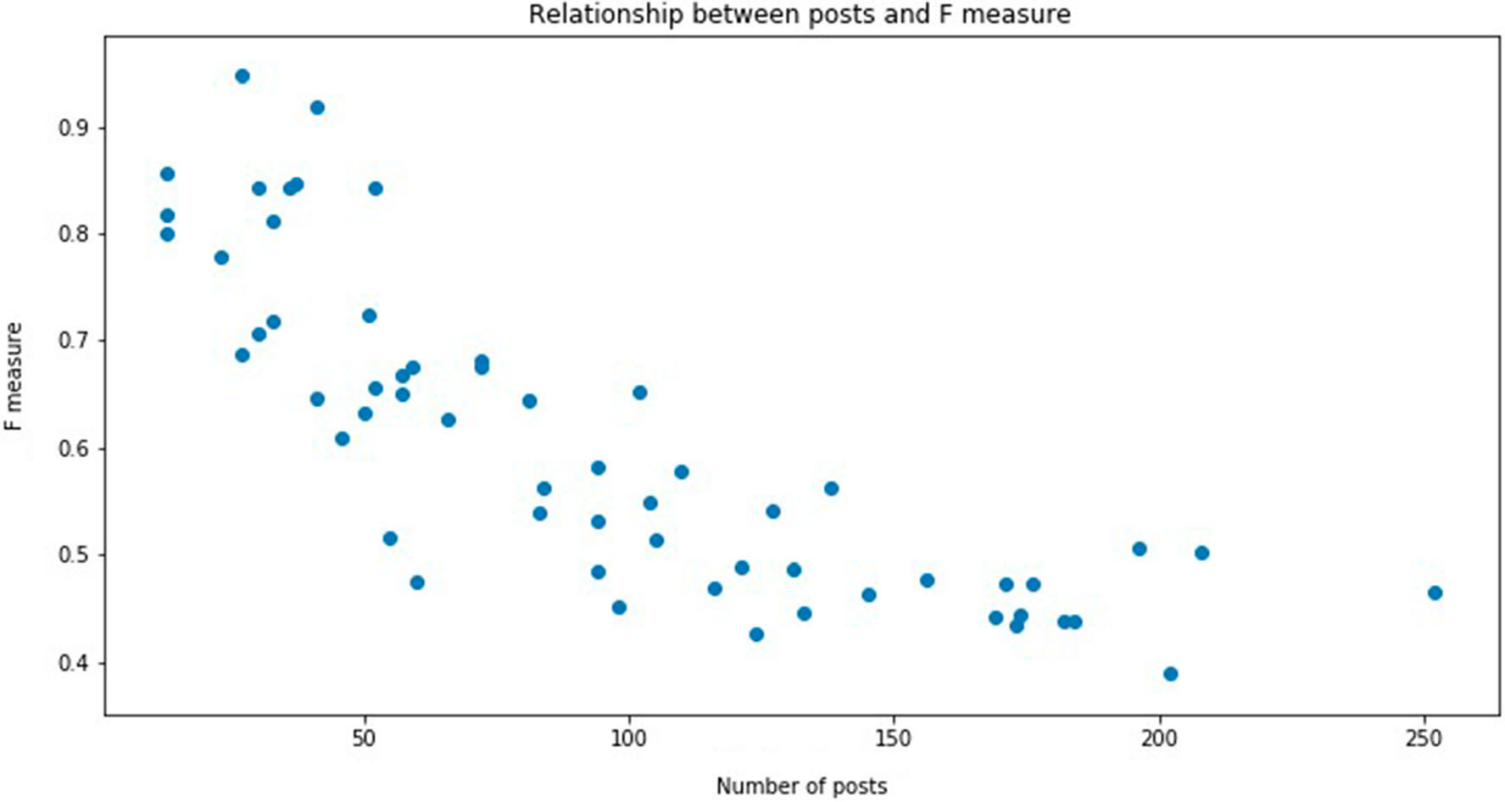
Relationship between number of posts and *F*-measure score

We get the best results in terms of *F* measure for sub-forum 6. It seems that the lower number of posts allows a more efficient semantic analysis and makes it easier for the model to find the threads a user finds interest in. A relevant observation is that as the number of posts increases in a sub-forum, the predictive results worsen. A qualitative interpretation is that it becomes harder to predict whether a user will post to a thread based on the semantic description of the content because it is contaminated with spurious unfiltered messages. In Fig. 10 we show the network graph corresponding to the month and sub-forum with worst performance results. We notice a large number of false positives. This led us to investigate further, so in Fig. 11 we show the scatter plot of the number of posts made in a unit period of time (month) versus the *F* measure score achieved by the neuro-semantic model in the same period. It appears that as the number of posts increases, the performance of ELCA model prediction decreases. As before, our interpretation is that the cause of this decrease is the increased heterogeneity of the semantic content in the thread, which becomes very noisy.

A way in which we could enhance the neuro-semantic model is to incorporate a discrimination behavior for users that will filter out posts that differ too much with the user semantic preference vector [41]. If we consider the temporal behavior of the *F* measure results within a sub-forum, the scores do not deviate much from the mean value, hence the LCA model is very robust in terms of temporal decay. We associate this behavior with parameter *a*. In this research, we set the value of $a=50$ without further search for an optimal setting. However, this parameter could also be optimized by the GA approach.

## Conclusions

This paper presents a neuro-semantic model of the content publication decisions of users in a web forum OSN at the microscopic level, i.e. the model predicts the specific decision of a user to post a message in a specific conversation thread of a sub-forum. We propose an extended leaky competition accumulator (ELCA) neural model that implements the competition of the diverse threads for the attention of the user as a dynamical process. Model parameter estimation was carried out by a genetic algorithm optimization process. To our knowledge, this is the first work where LCA parameters are estimated from data obtained from a social network content generation prediction in order to achieve optimal predictive performance. The revised literature contains rough qualitative settings of the parameters in order to study the emergent behavior according to theories of value based choice. On the other hand, we have not detected some well known choice phenomena like the preference reversals. More in detail analysis might uncover such phenomena in our problem domain.

Semantic similarity underlaying the attention mechanism is modeled by unsupervised topic analysis, thus it is fully automated. Results over the data extracted from a real life OSN are quite promising. Specifically the ELCA model improves greatly over standard machine learning approaches, namely random forest (RF) and support vector machines (SVM), using the same kind of semantic information as input features. Best and average F score of ELCA was 0.95 and 0.61, respectively, while for the RF and SVM best F score was 0.60 and 0.63, respectively, and the average F score was 0.19 and 0.21, respectively. The fundamental research into the likelihood maximization approaches to LCA parameter estimation is a priority for future works.

Further work will be directed to a deeper exploration into the fundamentals of Natural Language Processing (NLP) algorithms in order to improve the capture of the real meaning of the posted text documents, overcoming frequentist approaches to model the joint occurrence of words in a document [13]. Automatic ontology creation for a specific domain is a promising approach to tackle this problem. We will explore word embeddings as a very powerful modeling approach at the expense of interpretability.

Finally, another quite exciting research area is topic space metrics. Future work could be addressed to the definition of an adequate distance between multi-topic text vector representations allowing the extraction of the most valuable content generated by users. Besides, the approach developed in this work could be combined with other existing methods that capture topological features of the network looking for an improvement in prediction performance by such a hybrid system.

## Data Availability

Data used for the computational experiments will be available in zenodo.org after paper acceptance for publication.

## Code availability

Specific code for the computational pipeline will be publish with the data in zenodo.orf after paper acceptance for publication.
